# Correspondence

**Published:** 1850-11

**Authors:** William Ring

**Affiliations:** Buffalo


To the Editor of the Buffalo Medical Journal.

My name appeared as one of Seventeen Physicians who signed a Card which was published in the March number of your Journal, disapproving of the course pursued by the Professor of Obstetrics in a case of Demonstrative Midwifery at the Medical College in this city.

That protest styled the demonstration as "unprofessional in manner, and grossly offensive alike to morality and common decency." After hearing the evidence before a Court of Justice as to that transaction, I am satisfied that the conduct of the Professor *was not* such as deserved censure. Therefore I choose to withdraw my assent to such an imputation of his character and motives.

WILLIAM RING.

*Buffalo,* 1850.

